# Supplementary material for: FIGNL1 AAA+ ATPase remodels RAD51 and DMC1 filaments in pre-meiotic DNA replication and meiotic recombination
Source: Nat Commun. 2023 Oct 27;14:6857. doi: 10.1038/s41467-023-42576-w (PMC10611733; doi:10.1038/s41467-023-42576-w)
Supplement: Supplementary file 1 — Supplementary Information [file 41467_2023_42576_MOESM1_ESM.pdf]

FIGNL1 AAA+ ATPase remodels RAD51 and DMC1 filaments in pre-meiotic DNA replication and meiotic recombination

Masaru Ito, Asako Furukohri, Kenichiro Matsuzaki, Yurika Fujita, Atsushi Toyoda, and Akira Shinohara

Supplementary information

Supplementary Figure 1-10
Supplementary Table 1-2
Supplementary References

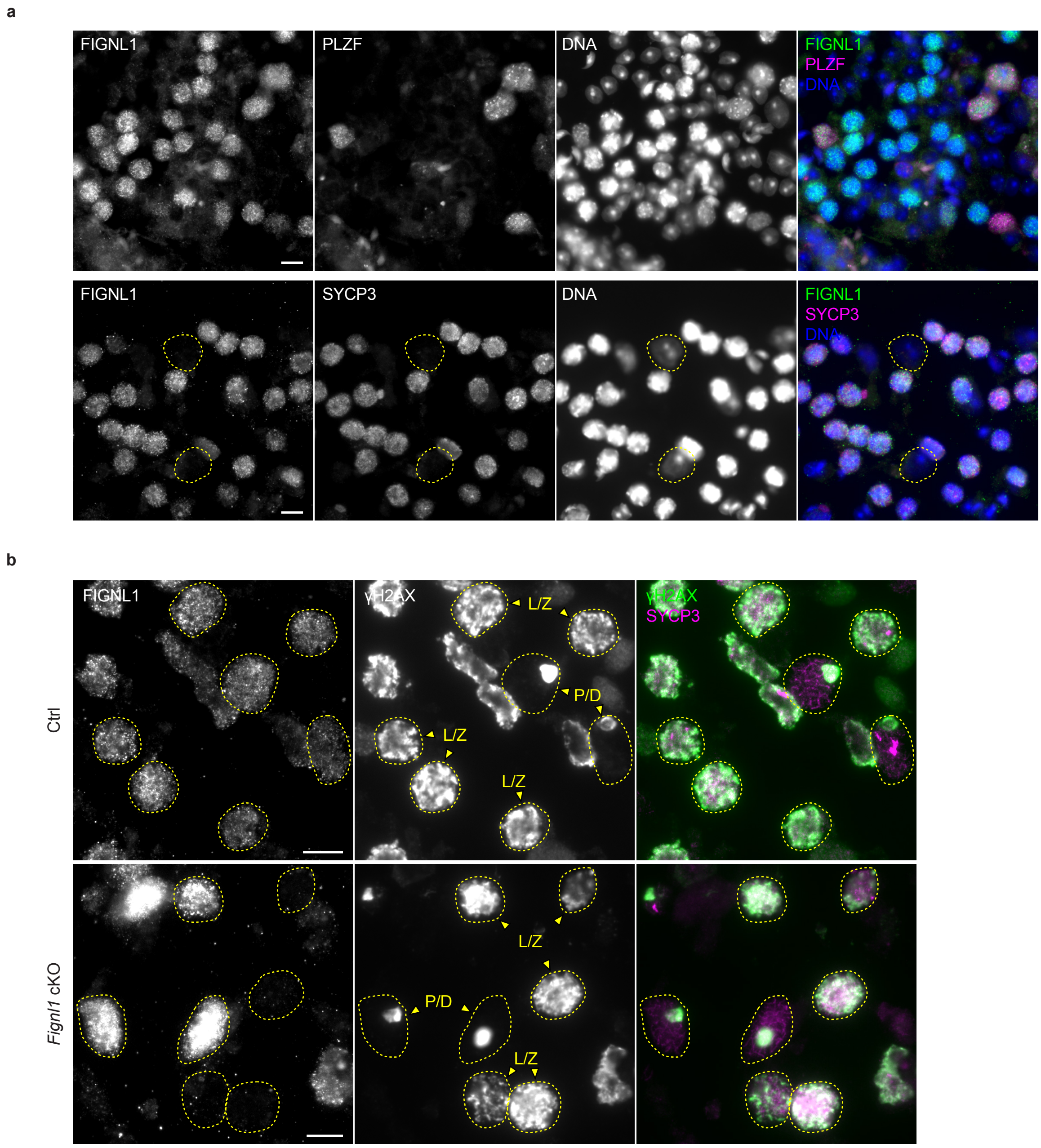

Supplementary Fig. 1. Depletion of FIGNL1 from early meiotic prophase I spermatocytes.
(**a**) Representative images of testicular cell squashes immunostained for FIGNL1 (green), PLZF (top, magenta) and SYCP3 (bottom, magenta), counterstained for DNA with DAPI (blue). Cells in yellow dotted circles with condensed DAPI bodies are Sertoli cells or round spermatids. Scale bars, 10 μm.
(**b**) Representative images of testicular cell squashes immunostained for FIGNL1 (green), γH2AX (blue), and SYCP3 (magenta) in Ctrl (*Fignl1*$^{+/+}$ *Stra8-Cre*$^{+}$) and *Fignl1* cKO (*Fignl1*$^{flox/\Delta}$ *Stra8-Cre*$^{+}$). SYCP3-positive prophase I spermatocytes were categorized into two by γH2AX staining: leptotene/zygotene cells (L/Z) and pachytene/diplotene cells (P/D) show pan-nuclear γ H2AX staining and the sex body-specific γH2AX staining, respectively. Scale bars, 10 μm.

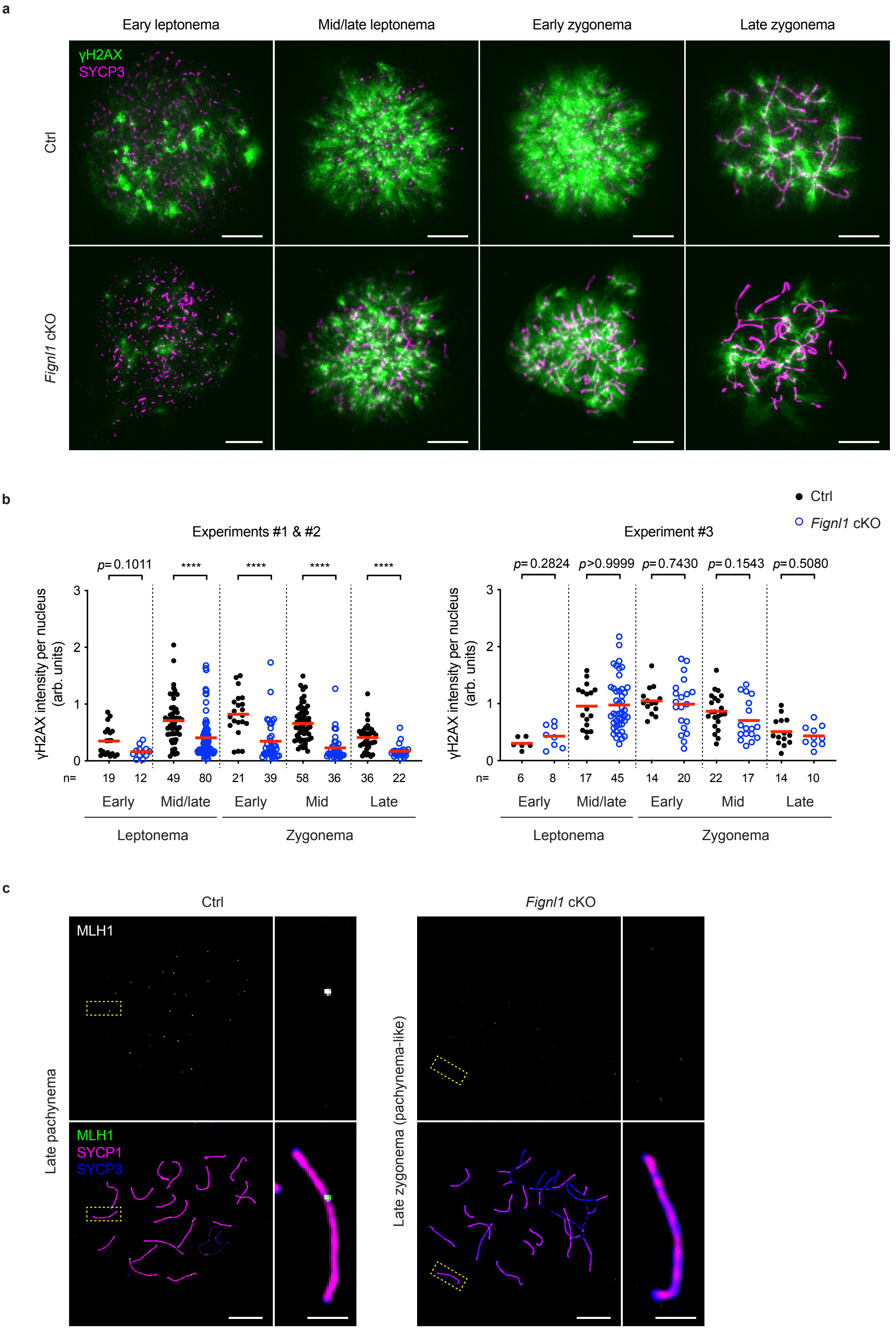

Supplementary Fig. 2. Deregulated meiotic recombination in *Fignl1* cKO spermatocytes.
(**a**) Representative images of spermatocyte-chromosome spreads immunostained for γH2AX (white in the top panels and green in the bottom panels) and SYCP3 (magenta) at indicated meiotic prophase I stages in Ctrl and *Fignl1* cKO.
(**b**) Quantification of γH2AX intensity at different meiotic prophase I stages in Ctrl (black circles) and *Fignl1* cKO (blue open circles). Two independent experiments showed reduced (left; pooled results from two animals of each genotype) and one experiment showed similar (right) levels of γH2AX intensity in *Fignl1* cKO spermatocytes compared to Ctrl. The red bars are means. The results of the two-tailed Mann-Whitney *U*-tests are indicated: ****$p \leq 0.0001$. The total number of cells analyzed is indicated below the graphs.
(**c**) Representative images of spermatocyte-chromosome spreads immunostained for MLH1 (white in the top panels and green in the bottom panels), SYCP1 (magenta), and SYCP3 (blue) at late pachynema in Ctrl and late zygonema (pachynema-like) in *Fignl1* cKO. The right panels are magnified images of regions with yellow dotted rectangles.
Genotypes of indicated animals are: Ctrl, *Fignl1*$^{+/+}$ *Stra8-Cre*$^{+}$; *Fignl1* cKO, *Fignl1*$^{flox/\Delta}$ *Stra8-Cre*$^{+}$.
Scale bars in (**a**) and (**c**), 10 μm and 2 μm for magnified images in (**c**).
Source data are provided as a Source Data file.

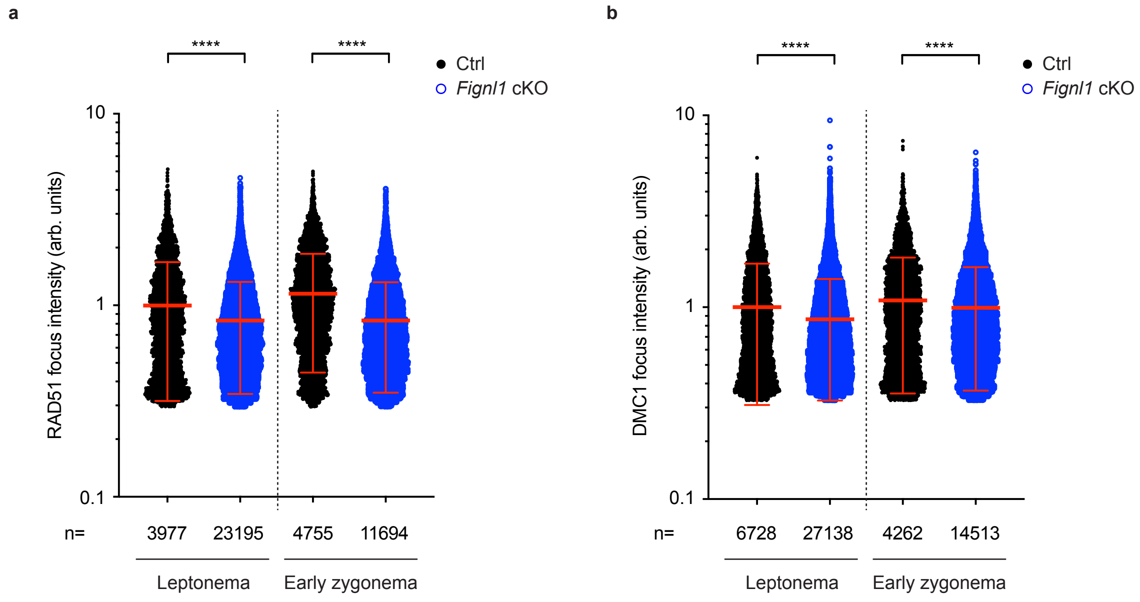

Supplementary Fig. 3. Reduced focus intensity of RAD51 and DMC1 in *Fignl1* cKO spermatocytes.
(**a**) Quantification of focus intensities of RAD51 at different meiotic prophase I stages in Ctrl (black circles) and *Fignl1* cKO (blue open circles). The red bars are means ± SDs. The total number of cells analyzed is 35 and 26 at leptonema and early zygonema in Ctrl, respectively; 54 and 30 at leptonema and early zygonema in *Fignl1* cKO, respectively.
(**b**) Quantification of focus intensities of DMC1 at different meiotic prophase I stages in Ctrl (black circles) and *Fignl1* cKO (blue open circles). The red bars are means ± SDs. The total number of cells analyzed is 64 and 22 at leptonema and early zygonema in Ctrl, respectively; 61 and 33 at leptonema and early zygonema in *Fignl1* cKO, respectively.
Genotypes of indicated animals are: Ctrl, *Fignl1*$^{+/+}$ *Stra8-Cre*$^{+}$; *Fignl1* cKO, *Fignl1*$^{flox/\Delta}$ *Stra8-Cre*$^{+}$. The results of the two-tailed Mann-Whitney *U*-test are indicated in the graphs: ****$p \leq 0.0001$. The total number of foci analyzed is indicated below the graphs.
Source data are provided as a Source Data file.

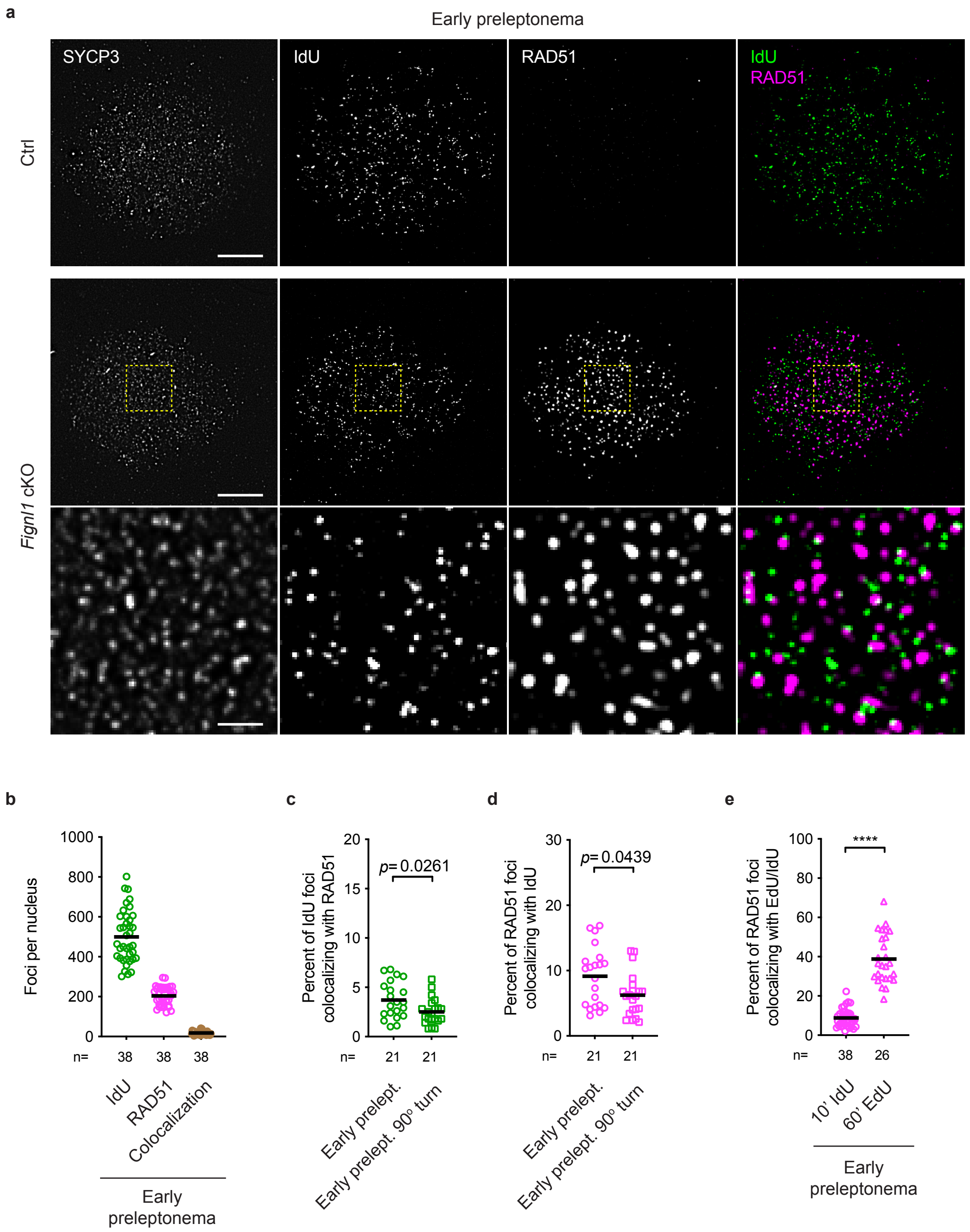

Supplementary Fig. 4. Infrequent localization of RAD51 at ongoing replication fork in preleptotene *Fignl1* cKO spermatocytes.
(**a**) Representative images of spermatocyte-chromosome spreads immunostained for IdU (green, 10' labeling), RAD51 (magenta), and SYCP3 at early preleptonema in Ctrl and *Fignl1* cKO. The bottom panels are magnified images of regions with yellow dotted rectangles. Scale bars, 10 μm for whole-nucleus images and 2 μm for magnified images.
(**b**) Quantification of focus numbers of IdU (green open circles), RAD51 (magenta open circles), and IdU-RAD51 colocalization (brawn open circles) at early preleptonema in *Fignl1* cKO. The black bars are means.
(**c** and **d**) Quantification of the frequency of IdU-RAD51 colocalization at early preleptonema in *Fignl1* cKO. The degrees of IdU foci colocalizing with RAD51 (**c**, green open circles) and RAD51 foci colocalizing with IdU (**d**, magenta open circles) are shown. The black bars are means. The colocalization frequency was measured with and without rotating the IdU image 90º clockwise relative to the RAD51 image.
(**e**) Comparison of the frequency of IdU-RAD51 (10' IdU labeling) and EdU-RAD51 (60' EdU labeling) colocalization at early preleptonema in *Fignl1* cKO. The degrees of RAD51 foci colocalizing with IdU (left, magenta open circles) and RAD51 foci colocalizing with EdU (right, magenta open triangles) are shown. The black bars are means.
Genotypes of indicated animals are: Ctrl, *Fignl1*$^{+/+}$ *Stra8-Cre*$^{+}$; *Fignl1* cKO, *Fignl1*$^{flox/\Delta}$ *Stra8-Cre*$^{+}$. The results of the two-tailed Mann-Whitney *U*-test are indicated in the graphs: ****$p \leq 0.0001$. The total number of cells analyzed is indicated below the graphs.
Source data are provided as a Source Data file.

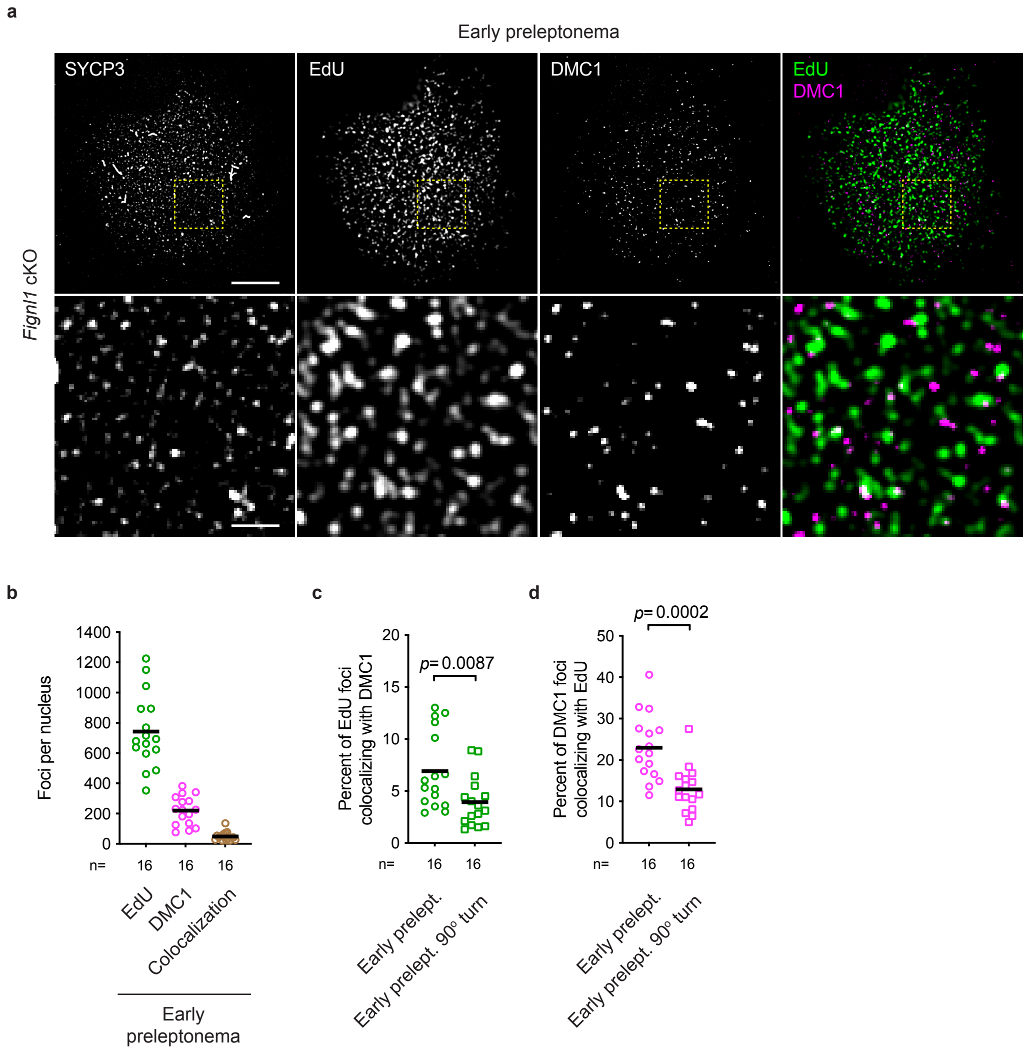

Supplementary Fig. 5. Infrequent localization of DMC1 at ongoing replication fork in preleptotene *Fignl1* cKO spermatocytes.

(**a**) Representative images of spermatocyte-chromosome spreads immunostained for EdU (green, 10' labeling), DMC1 (magenta), and SYCP3 at early preleptonema in Ctrl and *Fignl1* cKO. The bottom panels are magnified images of regions with yellow dotted rectangles. Scale bars, 10 μm for whole-nucleus images and 2 μm for magnified images.

(**b**) Quantification of focus numbers of EdU (green open circles), DMC1 (magenta open circles), and EdU-DMC1 colocalization (brawn open circles) at early preleptonema in *Fignl1* cKO. The black bars are means.

(**c** and **d**) Quantification of the frequency of EdU-DMC1 colocalization at early preleptonema in *Fignl1* cKO. The degrees of EdU foci colocalizing with DMC1 (**c**, green open circles) and DMC1 foci colocalizing with EdU (**d**, magenta open circles) are shown. The black bars are means. The colocalization frequency was measured with and without rotating the EdU image 90° clockwise relative to the DMC1 image.

Genotypes of indicated animals are: Ctrl, *Fignl1*$^{+/+}$ *Stra8-Cre*$^{+}$; *Fignl1* cKO, *Fignl1*$^{flox/\Delta}$ *Stra8-Cre*$^{+}$. The results of the two-tailed Mann-Whitney *U*-test are indicated in the graphs. The total number of cells analyzed is indicated below the graphs.

Source data are provided as a Source Data file.

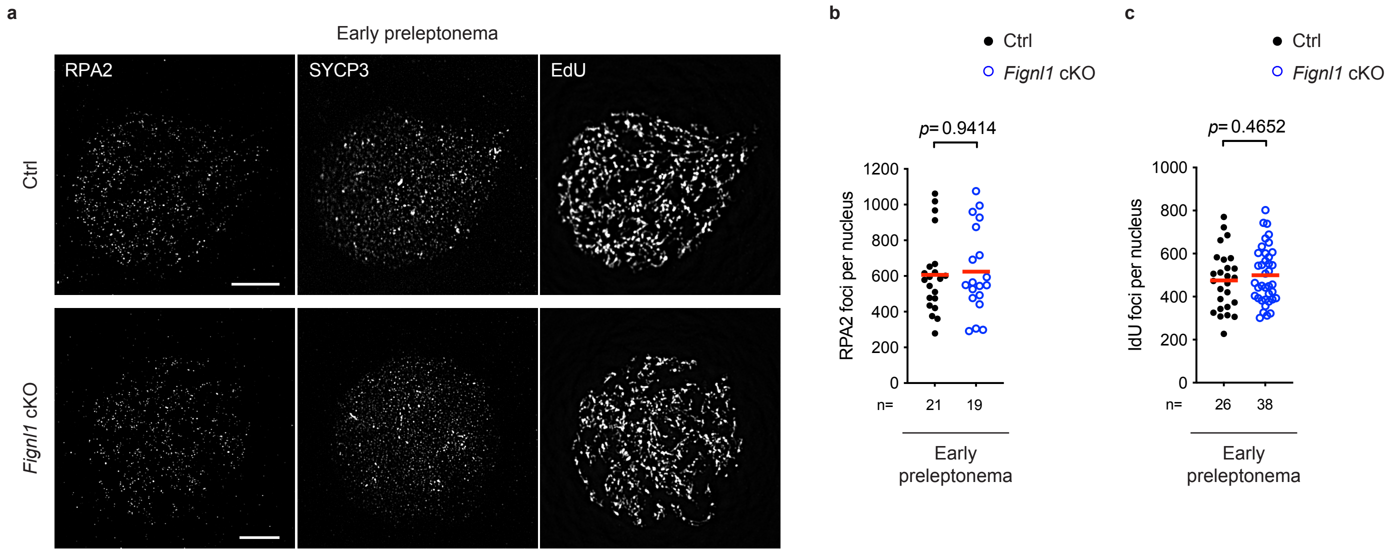

Supplementary Fig. 6. Largely normal pre-meiotic DNA replication in *Fignl1* cKO spermatocytes.
(**a**) Representative images of spermatocyte-chromosome spreads immunostained for RPA2 (left), SYCP3 (middle) and EdU (right, 60' labeling) at preleptonema in Ctrl and *Fignl1* cKO. Scale bars, 10 µm.
(**b**) Quantification of RPA2 focus numbers at early preleptonema in Ctrl (black circles) and *Fignl1* cKO (blue open circles). The red bars are means.
(**c**) Quantification of IdU focus numbers (10' IdU labeling) at early preleptonema in Ctrl (black circles) and *Fignl1* cKO (blue open circles). The red bars are means. Representative images of spermatocyte-chromosome spreads are shown in Supplementary Fig. **4a**.
Genotypes of indicated animals are: Ctrl, *Fignl1*$^{+/+}$ *Stra8-Cre*$^{+}$; *Fignl1* cKO, *Fignl1*$^{flox/\Delta}$ *Stra8-Cre*$^{+}$. The results of the two-tailed Mann-Whitney *U*-test are indicated in the graphs. The total number of cells analyzed is indicated below the graphs.
Source data are provided as a Source Data file.

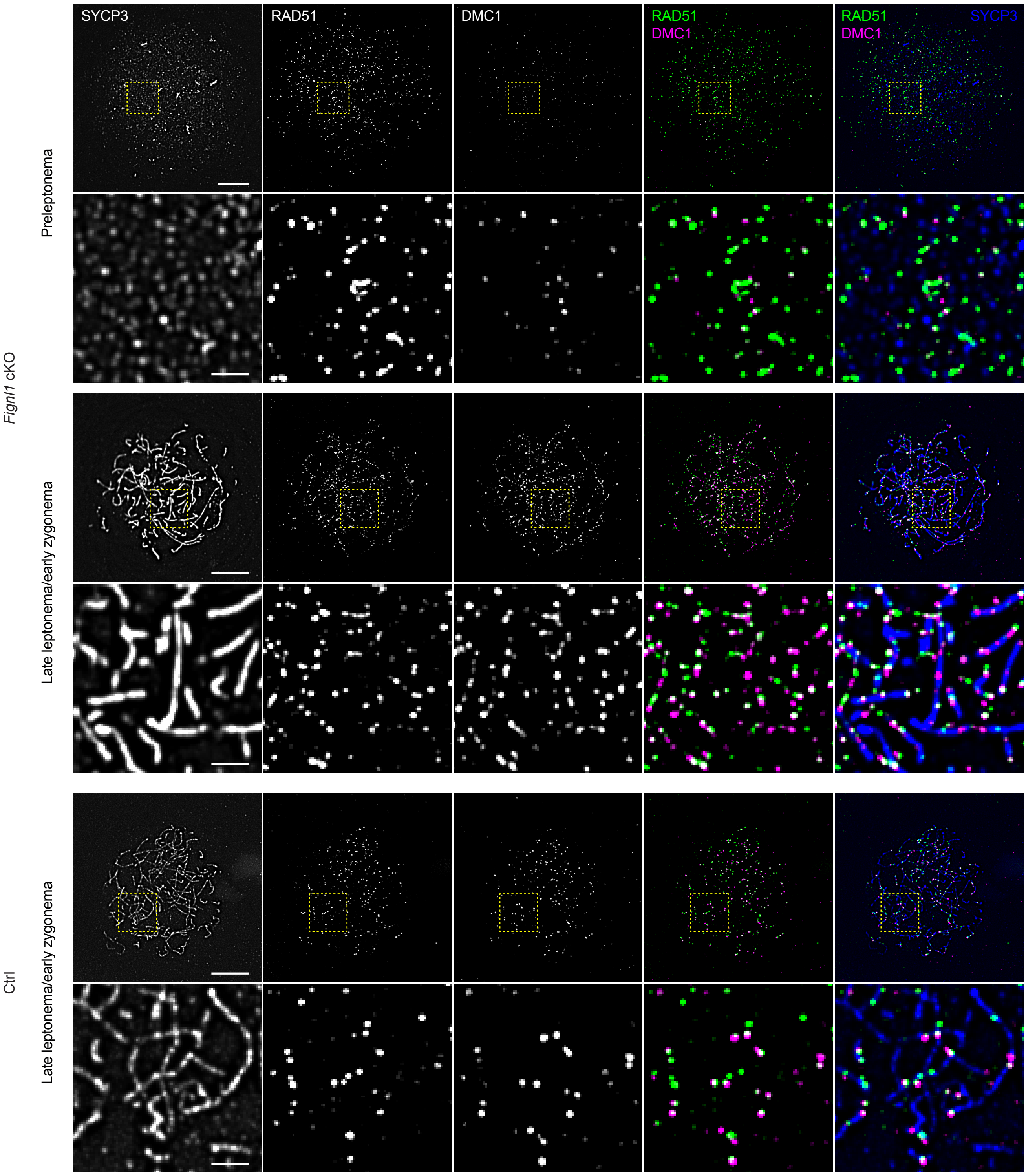

Supplementary Fig. 7. Uncoupled loading of RAD51 and DMC1 in *Fignl1* cKO spermatocytes.
Representative images of spermatocyte-chromosome spreads immunostained for RAD51 (green), DMC1 (magenta), and SYCP3 (blue) at indicated meiotic prophase I stages in Ctrl (*Fignl1*$^{+/+}$ *Stra8-Cre*$^{+}$) and *Fignl1* cKO (*Fignl1*$^{flox/\Delta}$ *Stra8-Cre*$^{+}$). The bottom panels are magnified images of regions with yellow dotted rectangles. Scale bars, 10 μm for whole-nucleus images and 2 μm for magnified images.

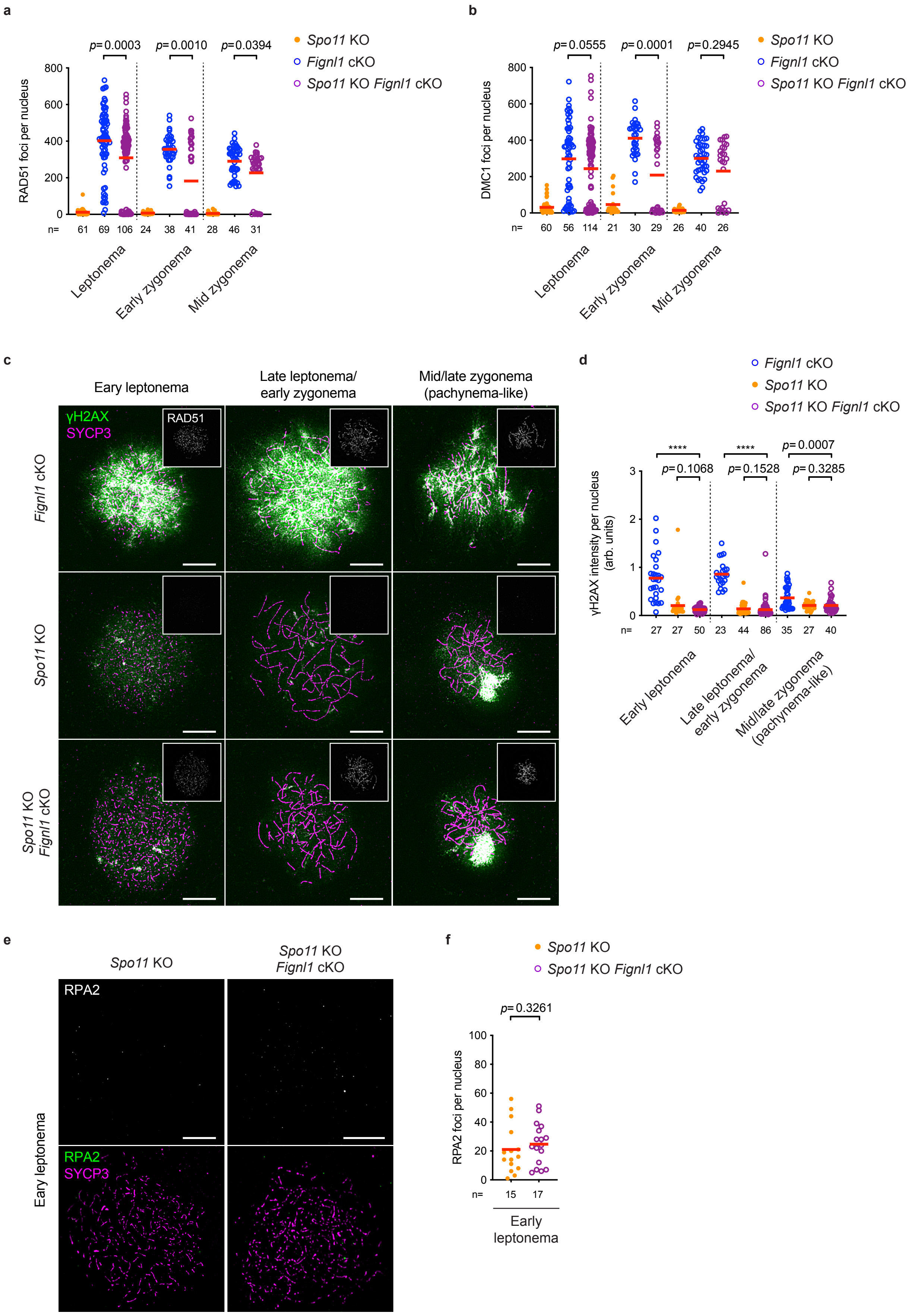

Supplementary Fig. 8. Accumulation of RAD51 and DMC1 with low levels of γH2AX and few RPA2 foci in *Spo11* KO *Fignl1* cKO spermatocytes.
(**a**) Quantification of RAD51 focus numbers at different meiotic prophase I stages in *Spo11* KO (orange circles), *Fignl1* cKO (blue open circles), and *Spo11* KO *Fignl1* cKO (purple open circles). The red bars are means.
(**b**) Quantification of DMC1 focus numbers at different meiotic prophase I stages in *Spo11* KO (orange circles), *Fignl1* cKO (blue open circles), and *Spo11* KO *Fignl1* cKO (purple open circles). The red bars are means.
(**c**) Representative images of spermatocyte-chromosome spreads immunostained for γH2AX (green), SYCP3 (magenta), and RAD51 (white in insets) at indicated meiotic prophase I stages in *Fignl1* cKO, *Spo11* KO, and *Spo11* KO *Fignl1* cKO.
(**d**) Quantification of γH2AX intensity at different meiotic prophase I stages in *Fignl1* cKO (blue open circles), *Spo11* KO (orange circles), and *Spo11* KO *Fignl1* cKO (purple open circles). The red bars are means.
(**e**) Representative images of spermatocyte-chromosome spreads immunostained for RPA2 (green) and SYCP3 (magenta) at early leptonema in *Spo11* KO and *Spo11* KO *Fignl1* cKO.
(**f**) Quantification of RPA2 focus numbers at early leptonema in *Spo11* KO (orange circles) and *Spo11* KO *Fignl1* cKO (purple open circles). The red bars are means.
Genotypes of indicated animals are: *Spo11* KO, *Spo11*$^{-/-}$ *Fignl1*$^{flox/Δ}$; *Fignl1* cKO, *Fignl1*$^{flox/Δ}$ *Stra8-Cre*$^{+}$; *Spo11* KO *Fignl1* cKO, *Spo11*$^{-/-}$ *Fignl1*$^{flox/Δ}$ *Stra8-Cre*$^{+}$. The results of the two-tailed Mann-Whitney *U*-test are indicated in the graphs: ****$p \leq 0.0001$. The total number of cells analyzed is indicated below the graphs.
Scale bars in (**c**) and (**e**), 10 μm.
Source data are provided as a Source Data file.

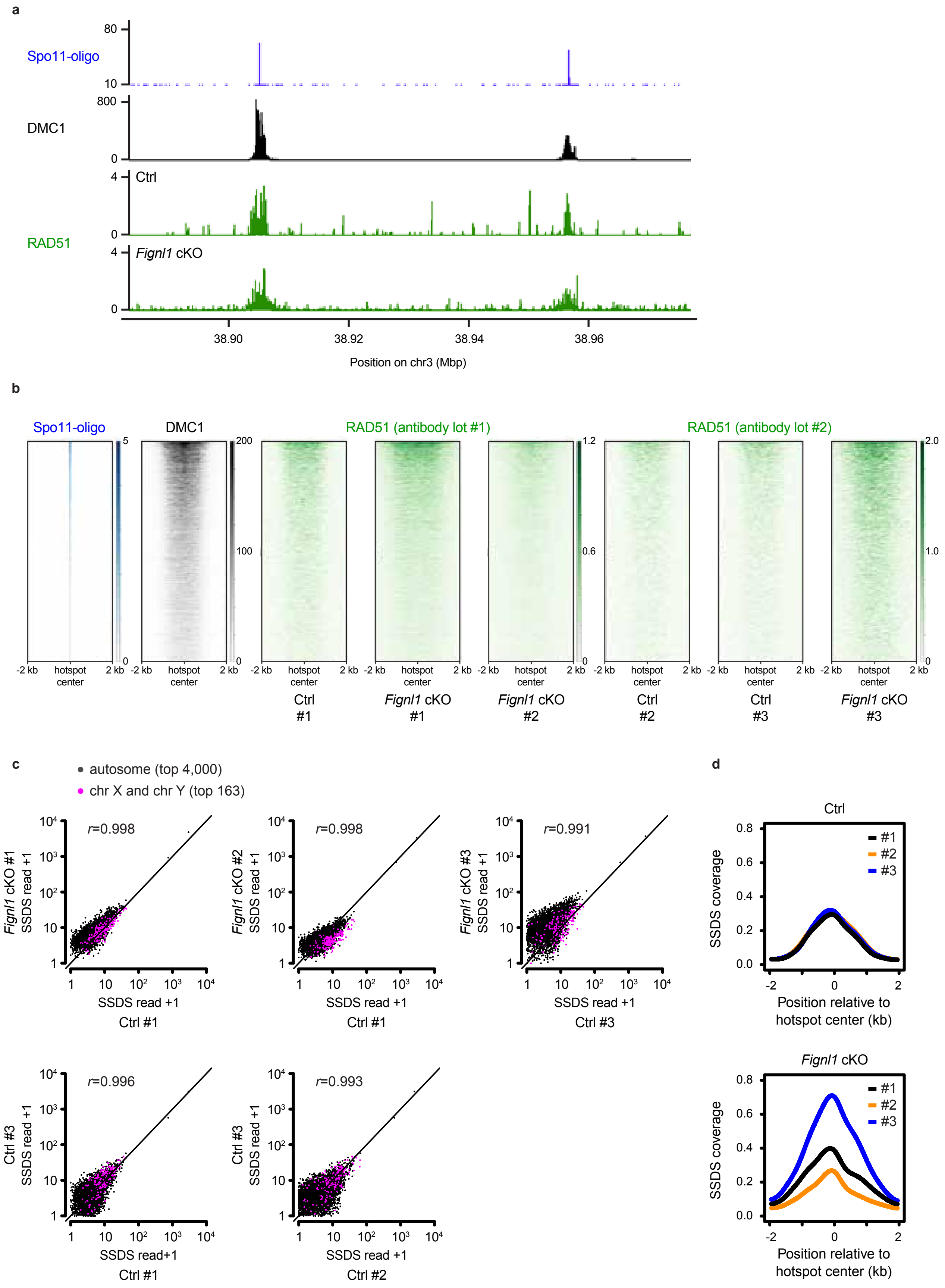

Supplementary Fig. 9. RAD51 binding at recombination sites in *Fignl1* cKO spermatocytes.

(**a**) RAD51 ChIP-SSDS signals (green) in Ctrl and *Fignl1* cKO with Spo11-oligo counts (blue) and DMC1 ChIP-SSDS signals (black) in wild-type at an indicated chromosomal region with multiple hotspots on mouse chromosome 3.

(**b**) The heatmap of RAD51 ChIP-SSDS signals (green) in Ctrl and *Fignl1* cKO and Spo11-oligo counts (blue) and DMC1 ChIP-SSDS signals (black) in wild-type, as shown in Fig. **6b**. Three mice of each genotype were analyzed and two RAD51 antibodies with different lot numbers were used for ChIP. The results of Ctrl #1 and *Fignl1* cKO #1 are shown in **a** and Fig. **6a**, **b**.

(**c**) Comparison of RAD51 ChIP-SSDS signals at meiotic recombination hotspots. Each spot represents the sum of RAD51 ChIP-SSDS signals around ±2,000 bp of one of the 4,000 most active meiotic recombination hotspots on autosomes (black) used for heatmap representation in **b** and Fig. **6b** and 163 active hotspots on sex chromosomes (magenta) with similar Spo11-oligo counts as the 4,000 active autosomal hotspots in wild-type [1]. Pearson's *r* for autosomal hotspots is also shown.

(**d**) Metaplots of RAD51 ChIP-SSDS signals around meiotic recombination hotspots. Smoothed RAD51 ChIP-SSDS signals in Ctrl (top) and *Fignl1* cKO (bottom) around ±2,000 bp of the 4,000 most active meiotic recombination hotspots on autosomes used for heatmap representation are shown. Three mice of each genotype were analyzed. Black, orange and blue lines represent the results of #1, #2 and #3, respectively.

Genotypes of indicated animals are: Ctrl, *Fignl1*$^{+/+}$ *Stra8-Cre*$^{+}$; *Fignl1* cKO, *Fignl1*$^{flox/\Delta}$ *Stra8-Cre*$^{+}$.

Source data are provided as a Source Data file.

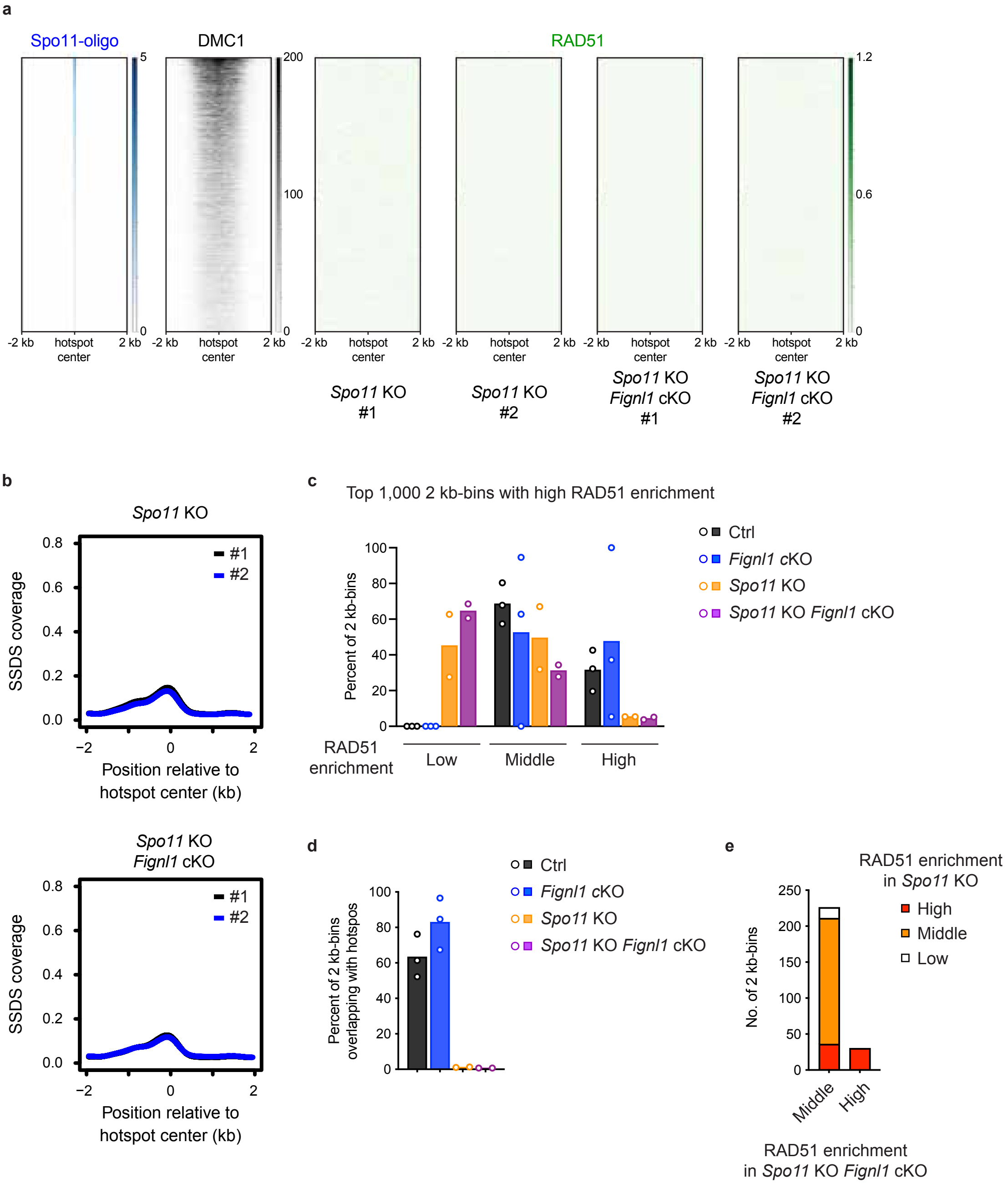

Supplementary Fig. 10. RAD51 binding in *Spo11* KO and *Spo11* KO *Fignl1* cKO spermatocytes.
(**a**) The heatmap of RAD51 ChIP-SSDS signals (green) in *Spo11* KO and *Spo11* KO *Fignl1* cKO and Spo11-oligo counts (blue) and DMC1 ChIP-SSDS signals (black) in wild-type, as shown in Fig. **6b**. Two mice of each genotype were analyzed.
(**b**) Metaplots of RAD51 ChIP-SSDS signals around meiotic recombination hotspots. Smoothed RAD51 ChIP-SSDS signals in *Spo11* KO (top) and *Spo11* KO *Fignl1* cKO (bottom) around ±2,000 bp of the 4,000 most active meiotic recombination hotspots on autosomes used for heatmap representation are shown. Two mice of each genotype were analyzed. Black and blue lines represent the results of #1 and #2, respectively.
(**c** and **d**) Comparison of RAD51 ChIP-SSDS profiles in the entire genome. Mouse genome was divided into non-overlapping 2-kb bins and top 1,000 bins with the highest RAD51 ChIP-SSDS signals were analyzed. The 1,000 bins were either categorized into three: high (>50), middle (50-20), and low (<20), based on sum RAD51 ChIP-SSDS signals in each bin (**c**) or two: overlapping with previously identified DMC1 ChIP-SSDS hotspots [2] and non-overlapping (**d**). The frequency of each category was plotted for each animal of Ctrl (black open circles), *Fignl1* cKO (blue open circles), *Spo11* KO (orange open circles), and *Spo11* KO *Fignl1* cKO (purple open circles). The bar graphs indicate means of three (Ctrl and *Fignl1* cKO) or two (*Spo11* KO and *Spo11* KO *Fignl1* cKO) mice of each genotype.
(**e**) Comparison of RAD51 ChIP-SSDS profiles in *Spo11* KO and *Spo11* KO *Fignl1* cKO. 262 bins with high or middle RAD51 ChIP-SSDS signals in both two of *Spo11* KO *Fignl1* cKO mice in (**c**) were categorized into high, middle, and low RAD51 ChIP SSDS signals in either of two *Spo11* KO mice by the same criteria as (**c**).
Genotypes of indicated animals are: Ctrl, *Fignl1*$^{+/+}$ *Stra8-Cre*$^{+}$; *Fignl1* cKO, *Fignl1*$^{flox/\Delta}$ *Stra8-Cre*$^{+}$; *Spo11* KO, *Spo11*$^{-/-}$ *Stra8-Cre*$^{+}$; *Spo11* KO *Fignl1* cKO, *Spo11*$^{-/-}$ *Fignl1*$^{flox/\Delta}$ *Stra8-Cre*$^{+}$.
Source data are provided as a Source Data file.

Supplementary Table 1. Primers used for genotyping used in this study.

**Primer**	**Sequence (5'->3')**
Fignl1 wild-type and flox allele, forward	TATCCATATTGGGTGTCTCCTGTTAC
Fignl1 wild-type and flox allele, reverse	TAGCACACACACACTGGATATAACT
Fignl1 deletion allele, forward	CCGAGTATCCATATTGGGTG
Fignl1 deletion allele, reverse	TGTCTGCTGTAGTCATGAGG
Stra8-Cre transgenic allele, forward	CTCCAAGGGGGTAAGGTGTAGC
Stra8-Cre transgenic allele, reverse	TTCCAGGGCGCGAGTTGATAG
Swsap1 wild-type allele, forward	GAGATATCGCTTTTGATTGGTC
Swsap1 wild-type allele, reverse	GAATTGAGGATGGGTACTGG
Swsap1 deletion allele, forward	CGGTTTCCATATGGGGATTGGTGGCGACGA
Swsap1 deletion allele, reverse	TGCCTTATTTCCTGATCCAGGCTAGCTGTC
Spo11 wild-type allele, forward	TGAGATACATGGAGGAAGATGG
Spo11 deletion allele, forward	CTGAGCCCAGAAAGCGAAGGAA
Spo11, common reverse	ATGTTAGTCGGCACAGCAGTAG

Supplementary Table 2. Antibodies used in this study.

Antibody	Source	Identifier	Application	Dilution
Rat anti-SYCP3 (Serum)	this study	N/A	IF	1:5,000
Mouse anti-SYCP3	Abcam	ab97672	IF	1:750
Rabbit anti-SYCP1	Novus Biologicals	NB300-229	IF	1:500
Mouse anti-MLH1	BD Pharmingen	51-1327QR	IF	1:50
Rabbit anti-MSH4	Abcam	ab58666	IF	1:200
Rabbit anti-RPA2	Abcam	ab76420	IF	1:200
Rat anti-RPA2	Cell Signaling	2208	IF	1:100
Mouse anti-γH2AX	Millipore	05-636	IF	1:500
Rabbit anti-DMC1	Santa Cruz	sc-22768	IF	1:200
Rabbit anti-RAD51	Santa Cruz	sc-8349	IF	1:100
Mouse anti-RAD51	Novus Biologicals	NB100-148	IF, ChIP	1:100
Mouse anti-BrdU	BD Biosciences	BD347580	IF	1:200
Mouse anti-PLZF	Santa Cruz	sc-28319	IF	1:100
Rabbit anti-FIGNL1	Proteintech	17604-1-AP	IF	1:100
Rabbit anti-FIGNL1	Proteintech	17604-1-AP	IB	1:1,000
Mouse anti-α tubulin	SIGMA	T6074	IB	1:3,000
Goat anti-mouse 488	Invitrogen	A-11029	IF	1:1,000
Goat anti-rabbit 488	Invitrogen	A-11034	IF	1:1,000
Goat anti-rat 488	Invitrogen	A-11006	IF	1:1,000
Goat anti-mouse 594	Invitrogen	A-11032	IF	1:1,000
Goat anti-rat 594	Invitrogen	A-11007	IF	1:1,000
Goat anti-rabbit 647	Invitrogen	A-21245	IF	1:500
Goat anti-rabbit IgG-HRP	Southern Biotech	4030-05	IB	1:10,000
Goat anti-mouse IgG-HRP	Thermo Scientific	31430	IB	1:10.000

IF, immunofluorescence; IB, immunoblotting.; ChIP, chromatin immunoprecipitation

Supplementary References

1. Lange, J. *et al.* The Landscape of Mouse Meiotic Double-Strand Break Formation, Processing, and Repair. *Cell* **167**, 695-708 e616 (2016).
2. Brick, K., Smagulova, F., Khil, P., Camerini-Otero, R.D. & Petukhova, G.V. Genetic recombination is directed away from functional genomic elements in mice. *Nature* **485**, 642-645 (2012).
