# Supplementary material for: FIGNL1 AAA+ ATPase remodels RAD51 and DMC1 filaments in pre-meiotic DNA replication and meiotic recombination
Source: Nat Commun. 2023 Oct 27;14:6857. doi: 10.1038/s41467-023-42576-w (PMC10611733; doi:10.1038/s41467-023-42576-w)
Supplement: Supplementary file 3 — Reporting Summary [file 41467_2023_42576_MOESM3_ESM.pdf]

nature portfolio

Corresponding author(s): Masaru Ito, Akira Shinohara

Last updated by author(s): Oct 6, 2023

Reporting Summary

Nature Portfolio wishes to improve the reproducibility of the work that we publish. This form provides structure for consistency and transparency in reporting. For further information on Nature Portfolio policies, see our Editorial Policies and the Editorial Policy Checklist.

Statistics

For all statistical analyses, confirm that the following items are present in the figure legend, table legend, main text, or Methods section.

n/a	Confirmed	
☐	☒	The exact sample size (*n*) for each experimental group/condition, given as a discrete number and unit of measurement
☐	☒	A statement on whether measurements were taken from distinct samples or whether the same sample was measured repeatedly
☐	☒	The statistical test(s) used AND whether they are one- or two-sided<br>*Only common tests should be described solely by name; describe more complex techniques in the Methods section.*
☒	☐	A description of all covariates tested
☒	☐	A description of any assumptions or corrections, such as tests of normality and adjustment for multiple comparisons
☐	☒	A full description of the statistical parameters including central tendency (e.g. means) or other basic estimates (e.g. regression coefficient) AND variation (e.g. standard deviation) or associated estimates of uncertainty (e.g. confidence intervals)
☐	☒	For null hypothesis testing, the test statistic (e.g. *F*, *t*, *r*) with confidence intervals, effect sizes, degrees of freedom and *P* value noted<br>*Give P values as exact values whenever suitable.*
☒	☐	For Bayesian analysis, information on the choice of priors and Markov chain Monte Carlo settings
☒	☐	For hierarchical and complex designs, identification of the appropriate level for tests and full reporting of outcomes
☐	☒	Estimates of effect sizes (e.g. Cohen's *d*, Pearson's *r*), indicating how they were calculated

Our web collection on statistics for biologists contains articles on many of the points above.

Software and code

Policy information about availability of computer code

Data collection	Images were acquired using a computer-assisted fluorescence microscope system (DeltaVision; Applied Precision) and image deconvolution was performed using softWoRx (6.5.2) software.
Data analysis	The softwares used in the study are: Adobe photoshop (23.2.2), Imaris (9.2.1), ImageJ/Fiji (Version 2.9.0), Graphpad Prism (Version 9.5.1), R (Version 4.2.2). A published bioinformatic pipeline for single-stranded DNA sequencing analysis is available at https://nf-co.re/ssds/dev or https://github.com/nf-core/ssds/tree/dev.

For manuscripts utilizing custom algorithms or software that are central to the research but not yet described in published literature, software must be made available to editors and reviewers. We strongly encourage code deposition in a community repository (e.g. GitHub). See the Nature Portfolio guidelines for submitting code & software for further information.

Data

Policy information about availability of data

All manuscripts must include a data availability statement. This statement should provide the following information, where applicable:

- Accession codes, unique identifiers, or web links for publicly available datasets
- A description of any restrictions on data availability
- For clinical datasets or third party data, please ensure that the statement adheres to our policy

The raw and processed sequencing data generated in this study have been deposited in the Gene Expression Omnibus (GEO) repository under accession number

GSE227944. Uncropped immunoblotting images and an unprocessed image of electrophoresis mobility shift assay are provided in a Source Data file. The raw data used in plots and graphs are all provided in a Source Data file. The raw microscopy images used for figures and quantification are available from corresponding authors upon request, because of a large number of images and the sufficient information (focus counts etc.) all provided in the Source Data file.

Research involving human participants, their data, or biological material

Policy information about studies with human participants or human data. See also policy information about sex, gender (identity/presentation), and sexual orientation and race, ethnicity and racism.

Reporting on sex and gender	NA
Reporting on race, ethnicity, or other socially relevant groupings	NA
Population characteristics	NA
Recruitment	NA
Ethics oversight	NA

Note that full information on the approval of the study protocol must also be provided in the manuscript.

Field-specific reporting

Please select the one below that is the best fit for your research. If you are not sure, read the appropriate sections before making your selection.

☒ Life sciences ☐ Behavioural & social sciences ☐ Ecological, evolutionary & environmental sciences

For a reference copy of the document with all sections, see nature.com/documents/nr-reporting-summary-flat.pdf

Life sciences study design

All studies must disclose on these points even when the disclosure is negative.

Sample size	Sample sizes were not predetermined using any statistical tests, and chosen based on past publications in the field: a minimum of three animals for testis weight and sperm counts to allow statistical analysis; a minimum of 90 cells for frequency analysis; a minimum of 10 nuclei at each meiotic prophase I stage for cytological analysis unless very few cells at a given stage were detectable. Given that the mutant mice used in this study showed very severe phenotypes in meiotic recombination and spermatogenesis, the chosen sample sizes were appropriate and justified. For example, please see the following past publications: Bondarieva et al., Proline-rich protein PRR19 functions with cyclin-like CNTD1 to promote meiotic crossing over in mouse. Nature Commun 11, 3101 (2020); Qiao et al., Antagonistic roles of ubiquitin ligase HEI10 and SUMO ligase RNF212 regulate meiotic recombination. Nature Genet 46, 194-199 (2014); Nore et al., TOPOVIBL-REC114 interaction regulates meiotic DNA double-strand breaks. Nature Commun 13, 7048 (2022).
Data exclusions	No data were excluded from the analysis.
Replication	At least two animals of each genotype were analyzed and similar results were obtained otherwise mentioned in the manuscript. Three independent experiments were performed for biochemical analysis.
Randomization	A pair or a set of animals that were either littermate or matched by age were randomly chosen for each experiment: after genotyping, animals with genotypes of interest were randomly chosen. For quantification of focus numbers and focus intensities, spermatocyte nuclei were randomly selected.
Blinding	Because the differences in phenotypes of control and mutant animals were clear and even blinded investigator would be able to distinguish the control from mutant samples, blinding is not relevant to our study. However, for cytological analysis of at least one pair of control and Fignl1 cKO animals, the investigator was blind to genotypes of the samples being analyzed.

Reporting for specific materials, systems and methods

We require information from authors about some types of materials, experimental systems and methods used in many studies. Here, indicate whether each material, system or method listed is relevant to your study. If you are not sure if a list item applies to your research, read the appropriate section before selecting a response.

Materials & experimental systems

n/a	Involved in the study
☐	☒ Antibodies
☒	☐ Eukaryotic cell lines
☒	☐ Palaeontology and archaeology
☐	☒ Animals and other organisms
☒	☐ Clinical data
☒	☐ Dual use research of concern
☒	☐ Plants

Methods

n/a	Involved in the study
☐	☒ ChIP-seq
☒	☐ Flow cytometry
☒	☐ MRI-based neuroimaging

Antibodies

Antibodies used

All primary and secondary antibodies used in this study are listed in Supplementary table.

Validation

The rat anti-SYCP3 serum were validated by immunostaining of spermatocyte chromosome spreads from wild-type testes and comparing its staining pattern with known staining pattern of the protein in spermatocyte chromosome spreads. The following commercially available antibodies have been validated by the manufacturers and past publications: Mouse anti-SYCP3 (Abcam, ab97672) and Rabbit anti-RAD51 (Santa Cruz, sc-8349), Matsuzaki et al., Human RAD51 paralogue SWSAP1 fosters RAD51 filament by regulating the anti-recombinase FIGNL1 AAA+ ATPase. Nature Commun 10, 1407 (2019); Rabbit anti-SYCP1 (Novus Biologicals, NB300-229), Sun and Handel, Regulation of the meiotic prophase I to metaphase I transition in mouse spermatocytes. Chromosoma 117, 471-485 (2008); Rabbit anti-MSH4 (Abcam, ab58666), Rabbit anti-DMC1 (Santa Cruz, sc-22768) and Mouse anti-γH2AX (Millipore, 05-636), Bondarieva et al., Proline-rich protein PRR19 functions with cyclin-like CNTD1 to promote meiotic crossing over in mouse. Nature Commun 11, 3101 (2020); Rabbit anti-RPA2 (Abcam, ab76420), Rao et al., A SUMO-ubiquitin relay recruits proteasomes to chromosome axes to regulate meiotic recombination. Science 355, 6323 (2017); Rat anti-RPA2 (Cell Signaling, 2208), Zelazowski et al., Age-dependent alterations in meiotic recombination cause chromosome segregation errors in spermatocytes. Cell 171, 601-614 (2017). The following commercially available antibodies were validated by the manufacturers and also by immunostaining of spermatocyte chromosome spreads or testicular cell squashes from wild-type testes as described above: Mouse anti-MLH1 (BD Pharmingen, 51-1327QR), https://www.bdbiosciences.com/en-us/products/reagents/microscopy-imaging-reagents/immunohistochemistry-reagents/purified-mouse-anti-mlh-1-with-control.551091; Mouse anti-RAD51 (Novus Biologicals, NB100-148), https://www.novusbio.com/products/rad51-antibody-14b4_nb100-148; Mouse anti-BrdU (BD Biosciences, BD347580), https://www.bdbiosciences.com/en-us/products/reagents/flow-cytometry-reagents/clinical-discovery-research/single-color-antibodies-ruo-gmp/purified-mouse-anti-brdu.347580; Mouse anti-PLZF (Santa Cruz, sc-28319), https://www.scbt.com/p/plzf-antibody-d-9. Rabbit anti-FIGNL1 antibody (Proteintech,17604-1-AP) was validated by the manufacturer (https://www.ptglab.com/products/FIGNL1-Antibody-17604-1-AP) and by immunostaining of testicular cell squashes from wild-type and Fignl1 cKO testes with negative staining specifically in Fignl1 cKO cells. Mouse anti-α tubulin (SIGMA, T6074) was validated by the manufacture (https://www.sigmaaldrich.com/JP/ja/product/sigma/t6074) and by immunoblotting of testis extracts and comparing its electrophoretic mobility with known electrophoretic mobility of the protein.

Animals and other research organisms

Policy information about studies involving animals; ARRIVE guidelines recommended for reporting animal research, and Sex and Gender in Research

Laboratory animals

Mus musculus mouse strains with Fignl1-flox, Stra8-Cre, Swsap1-KO and Spo11-KO alleles with the C57BL/6 background were used for experiments at 8-31 weeks of age. Mus musculus mouse strains with CAG-Cre allele with the C57BL/6 background and Spo11-KO allele with a mixed background of C57BL/6 and129/SvJ were used for breeding.

Wild animals

The study did not involve wild animals.

Reporting on sex

The study includes analysis only in males (spermatocytes) because of inefficient conditional knockout of Fignl1 gene by Stra8-Cre-mediated excision in females (oocytes).

Field-collected samples

The study did not involve samples collected from the fields.

Ethics oversight

The care and use of mice in this study were performed under the guideline for the proper conduct of animal experiments (Society Council of Japan). These procedures were approved by the Institutional Animal Care Committee at Institute for Protein Research, Osaka University (approval ID; 25-03-0).

Note that full information on the approval of the study protocol must also be provided in the manuscript.

Plants

Seed stocks

Report on the source of all seed stocks or other plant material used. If applicable, state the seed stock centre and catalogue number. If plant specimens were collected from the field, describe the collection location, date and sampling procedures.

Novel plant genotypes

Describe the methods by which all novel plant genotypes were produced. This includes those generated by transgenic approaches, gene editing, chemical/radiation-based mutagenesis and hybridization. For transgenic lines, describe the transformation method, the

number of independent lines analyzed and the generation upon which experiments were performed. For gene-edited lines, describe the editor used, the endogenous sequence targeted for editing, the targeting guide RNA sequence (if applicable) and how the editor was applied.

Authentication

Describe any authentication procedures for each seed stock used or novel genotype generated. Describe any experiments used to assess the effect of a mutation and, where applicable, how potential secondary effects (e.g. second site T-DNA insertions, mosiacism, off-target gene editing) were examined.

ChIP-seq

Data deposition

☒ Confirm that both raw and final processed data have been deposited in a public database such as GEO.

☐ Confirm that you have deposited or provided access to graph files (e.g. BED files) for the called peaks.

Data access links<br>*May remain private before publication.*	GSE227944 (https://www.ncbi.nlm.nih.gov/geo/query/acc.cgi?acc=GSE227944)
Files in database submission	RAD51 ChIP-SSDS, control rep1; control rep2; control rep3<br>RAD51 ChIP-SSDS, Fignl1 cKO rep1; Fignl1 cKO rep2; Fignl1 cKO rep3<br>RAD51 ChIP-SSDS, Spo11 KO rep1; Spo11 KO rep2<br>RAD51 ChIP-SSDS, Spo11 KO Fignl1 cKO rep1; Spo11 KO Fignl1 cKO rep2
Genome browser session<br>(e.g. UCSC)	Integrated Genome Browser 9.1.10 (UCSC)

Methodology

Replicates	Three independent animals were analyzed for both control and Fignl1 cKO mice.
Sequencing depth	Each sequencing sample was run with a 50 bp pf paird-end, and generated >65 million reads with >80% of uniquely mapped reads.
Antibodies	Mouse anti-RAD51 (Novus Biologicals, NB100-148)
Peak calling parameters	A published bioinformatic pipeline (https://nf-co.re/ssds/dev) was run for the resulting reads for mapping and the identification of single-stranded sequences. Because the aim of the analysis was to test whether or not RAD51 is enriched around previously defined DSB hotspots, peak call was not necessary and not carried out.
Data quality	FDRs for previously defined DSB hotspots (Brick et al., 2012, PMID: 22660327) are 4.5, 4.8, and 4.7% for control rep1, rep2, and rep3, respectively, and 5.6, 3.7, and 10.5% in Fignl1 cKO rep1, rep2, and rep3, respectively. Given that heatmap representation in the figures clearly showed enrichment of RAD51 around previously defined DSB hotspots, the data quality is appropriate. Although no clear enrichment of RAD51 was observed around DSB hotspots in Spo11 KO and Spo11 KO Fignl1 cKO samples as expected, those samples were collected and processed in the same condition as control and Fignl1 cKo samples.
Software	FastQC, Trimgalore, BWA, Picard, Samtools, Bedtools, Deeptools and MultiQC softwares were used according to a published bioinformatic pipeline (https://nf-co.re/ssds/dev).
